# Comparative study of the efficacy of tamoxifen and aromatase inhibitors among breast cancer patients at Kenyatta National Hospital

**DOI:** 10.1186/s43046-025-00309-8

**Published:** 2025-08-04

**Authors:** Henry Gachoki Macharia, Amsalu Degu

**Affiliations:** Department of Pharmaceutics and Pharmacy Practice, School of Pharmacy and Health Sciences, United States International University-Africa, Nairobi, Kenya, Nairobi, Kenya

**Keywords:** Aromatase inhibitors, Tamoxifen, Comparative efficacy, Kenyatta National Hospital

## Abstract

**Background:**

Aromatase inhibitors have demonstrated superior outcomes compared to tamoxifen in various studies. However, research in Africa, including Kenya, where breast cancer mortality rates are disproportionately high, is lacking.

**Objectives:**

The study aimed to assess the comparative efficacy of tamoxifen and aromatase inhibitors among hormone receptor-positive breast cancer patients at Kenyatta National Hospital.

**Methods:**

A retrospective cohort study was conducted at the Oncology Department of Kenyatta National Hospital, involving all eligible hormone receptor-positive breast cancer patients treated in the facility between 1st January 2019 to 31st December 2022. The study was hospital-based and used a data abstraction tool to collect data from the patients’ medical records. The data obtained was then analyzed using SPSS version 25 and Kaplan–Meier analysis was used to estimate the median survival time. Cox regression analysis was employed to determine whether there was a significant association between the variables. The collected data was presented in the form of frequency tables and graphs.

**Results:**

In our study, aromatase inhibitors consistently demonstrated superior outcomes compared to tamoxifen across various parameters. Specifically, aromatase inhibitors showed a lower incidence of disease progression (24% vs. 29.7%), a higher rate of complete radiological response (24% vs. 13.5%), and a reduced likelihood of developing distant metastasis while on treatment, coupled with a lower mortality rate (40% vs. 48.0%). Additionally, the median survival time for patients receiving aromatase inhibitors was notably longer at 49.0 months compared to 42.0 ± 3.6 months for those on tamoxifen (*P* = 0.410). Similarly, the aromatase inhibitor group exhibited a more extended median metastasis-free survival time (42.0 months vs. 30.0 ± 1.4 months, *P* = 0.056) and a more favorable survival time from metastasis to death (8 ± 0.6 months vs. 6 ± 0.8 months in the tamoxifen group, *P* = 0.142).

**Conclusion:**

These findings collectively suggest a consistent trend towards improved treatment outcomes with aromatase inhibitors compared to tamoxifen. The observed reduction in mortality rates among aromatase inhibitor–treated patients highlights their potential clinical benefit, with superior overall survival and disease progression.

## Background

“Breast cancer” is the most prevalent type of cancer globally [1–3]. However, due to better treatment regimens in the past decades, the mortality rate in most countries worldwide is declining [4].

With the projected increase in cancer incidence across the board, breast cancer is projected to rise significantly worldwide [1, 5–8]. Currently, Africa has the lowest number of cancers among all the other continents in the world, with the highest mortality rates [9].

Despite the high prevalence of breast cancer in developed countries, the mortality rates are way lower compared to Africa. The biggest factor that has contributed to the reduced mortality rates of breast cancer is better chemotherapy [10]. The gold standard of hormonal therapy is tamoxifen(both in post-menopausal and premenopausal) and aromatase inhibitors (only in post-menopausal women). Despite this therapy having already been established, Africa still has the highest mortality and the lowest prevalence.

Tamoxifen was first approved to be used in the management of breast cancer in 1977. It showed significant clinical benefits in improving survival outcomes and reducing recurrence rates [11].

Previous studies revealed that aromatase inhibitors increased overall survival compared to tamoxifen among early-stage breast cancer patients [12–14]. Other studies also reported that aromatase had a better overall survival than tamoxifen [13]. In another recent study, it was shown that in premenopausal women with ovarian suppression being treated by aromatase inhibitors for breast cancer, the recurrence rates were lower compared to the group being treated with tamoxifen [14]. Letrozole is clearly the superior agent for reducing the progression and relapse of breast cancer. Anastrozole, despite not being as effective as letrozole, is still superior to tamoxifen in halting progression and reducing recurrence and relapse [15, 16]. Despite this, studies on the African population were limited in comparing the efficacy of aromatase enzyme inhibitors and tamoxifen. Therefore, this research study aimed to investigate which of the hormonal therapies approved as first-line hormonal therapies best reduce mortality and improve clinical outcomes in treating hormone receptor-positive breast cancer patients in the study setting.

## Methods

### Study design, setting, and period

A retrospective cohort study was done at Kenyatta National Hospital (KNH) to determine the comparative efficacy of tamoxifen and aromatase enzyme inhibitors in the management of hormone receptor-positive breast cancer patients. The facility is a level six hospital based in Kenya along Hospital Road, Nairobi. It is the largest hospital in Kenya. This study was carried out from 1 st January 2023 to 28th February 2024 at the Oncology Department of KNH.

### Eligibility criteria

#### Inclusion criteria

- Medical records of women aged 18 years and above with a confirmed diagnosis of estrogen receptors (ER) or progesterone receptors (PR) and ER-positive breast cancer who received treatment with either tamoxifen or aromatase inhibitors between 1 st January 2019 and 31 st December 2022.
- Patients who were on continuous treatment with tamoxifen or aromatase inhibitors for at least 1 year.
- Patients whose other cancer treatment modalities (e.g., surgery, chemotherapy, radiotherapy) were comparable between the tamoxifen and aromatase inhibitor groups.

#### Exclusion criteria

- Medical records with incomplete data regarding the breast cancer diagnosis or treatment regimen.
- Breast cancer patients who did not receive tamoxifen or aromatase inhibitors as part of their treatment.
- Patients with documented concurrent primary tumors other than breast cancer.
- Patients who received sequential hormonal therapy involving both tamoxifen and aromatase inhibitors during the treatment period.
- Patient with human epidermal growth factor receptor 2 (HER2) positive and ER and PR negative breast cancer

### Sample size

All eligible breast cancer patients treated in the facility from 1 st January 2019 to 31 st December 2022 were included in the study. A total of 200 breast cancer patients who had received tamoxifen or aromatase inhibitors were sourced from the Health Information Department of the Hospital. However, 138 medical records of breast cancer patients were excluded from the study due to the lack of complete medical records. After the exclusion of incomplete medical records, a total of 62 medical records of breast cancer patients were included in the study.

### Research instruments

A data abstraction instrument was employed to gather data from patients’ medical records. This tool encompassed vital details, including treatment protocols utilized, sociodemographic, clinical characteristics, and parameters to measure survival outcomes (median survival, mortality, and treatment response). To assess the reliability and validity of the data collection tool, a pre-test study was conducted on 5% of the total sample size. This preliminary study helped identify any necessary adjustments required for the data collection tool before its implementation in the main study.

### Data collection techniques

The Health Information Department of Kenyatta National Hospital was contacted to access the patients’ medical records. A well-organized data abstraction form was used to review the pertinent patient charts. This assessment covered the patient’s socio-demographics, histological subtype, and clinical characteristics, including the types of treatment modalities used, time of diagnosis, disease progression, median survival time, and mortality. For patients who died in the hospital, confirmation of death was based on documentation from medical records and death certificates. However, for patients who died outside the hospital, we were unable to confirm their death due to incomplete or outdated contact information. Assessment of the tumor response was measured based on the report of the computed tomography (CT) scan imaging. In this study, all patients received adjuvant hormonal therapy, and no neoadjuvant treatment was administered. Hence, tumor response refers to the changes in the tumor size after the administration of the adjuvant treatment.

### Study endpoints

The primary endpoints of this study included the incidence of disease progression, rate of complete response, and mortality rate among breast cancer patients treated with tamoxifen versus aromatase inhibitors. Secondary endpoints consisted of median overall survival time, metastasis-free survival time, and survival time from metastasis to death. Specifically, the study measured disease progression as the proportion of patients whose cancer advanced during treatment, complete response as the percentage achieving full tumor remission, and mortality as the proportion of deaths recorded during the study period. Additionally, time-to-event outcomes such as median survival and metastasis-free survival were evaluated to compare long-term efficacy between the two treatment groups.

### Data analysis

The data were entered and analyzed using SPSS version 25 software. Kaplan–Meier analysis and Cox regression were employed to estimate the median survival times and the predictors of survival, respectively. The findings of the study were presented as frequency tables, percentages, graphs, and median survival time.

## Results

### Socio-demographic characteristics of breast cancer patients

The mean age of patients in this study was 53.0 ± 9.2 years (range 32–74 years). Most of the patients were married (85.5%), followed by widowed patients at 11.3%, with the least being single patients at 3.2%. For the level of education, the majority of the patients had tertiary (41.9%), secondary (29.0%), and primary (22.6%) education. The majority of the patients (54.8%) were post-menopausal women (Table 1).

**Table 1:** Socio-demographic characteristics of patients (*n* = 62)

**Variable**	**Frequency**	**Percent (%)**
Age range (32–74 years)		
Marital status		
Single	2	3.2
Widowed	7	11.3
Married	53	85.5
Level of education		
Illiterate	4	6.5
Primary	14	22.6
Secondary	18	29.0
Tertiary	26	41.9
Employment status		
Retired	15	9.9
Employed	23	37.1
Private employee	12	19.4
Unemployed	10	16.1
Other	16	25.8
Menopausal status		
Premenopausal	28	45.2
Post-menopausal	34	54.8

### Clinical characteristics of breast cancer patients

The majority of patients in this study had stage IV breast cancer (38.7%). This was followed by those with stage III cancer (32.3%) and lastly those with stage II cancer (29.0%). In this study, the majority of patients (80.7%) had tumors that were both ER-positive and PR-positive. A smaller proportion of patients had tumors that were ER-positive but PR-negative (16.1%), while only 3.2% had PR-positive but ER-negative tumors. The study showed that 80.7% of patients were classified as having Luminal A tumors. The remaining 16.1% were classified as Luminal B. All the patients in this study had invasive ductal carcinoma. A major proportion of patients did not have any comorbidities at diagnosis (77.4%), while 22.6% of patients had comorbidities (Table 2).

**Table 2:** Clinical characteristics of study participants (*n* = 62)

Variable	Frequency	Percent (%)
Stage of cancer		
Stage II	18	29
Stage III	20	32.3
Stage IV	24	38.7
Hormone receptor status		
ER-positive and PR-positive	50	80.7
ER-positive and PR-negative	10	16.1
PR-positive and ER-negative	2	3.2
Luminal classification		
Luminal-A (ER-positive, PR-positive, HER2-negative, and low Ki-67)	50	
Luminal-B(ER-positive, PR-negative, HER2-negative, and high Ki-67)	10	
Histological type		
Invasive ductal carcinoma	62	100
Invasive lobular carcinoma	0	0
Papillary carcinoma	0	0
Co-morbidity		
Present	14	22.6
Absent	48	77.4
Types of co-morbidity		
COPD	5	42.9
Osteoporosis	3	21.4
Diabetes mellitus	2	14.3
Other	4	28.6

*Other:* hypertension, toxic goitre, deep vein thrombosis, flaccid paralysis,* COPD* chronic obstructive pulmonary disease

### Treatment modalities administered to breast cancer patients

During the study period, a total of 300 breast cancer cases were identified. Among these, 200 patients were initiated on hormonal therapy. This represents 66.7% of the total breast cancer cases during that time.

A greater majority of patients in this study were treated with tamoxifen (59.7%) while the rest were treated with aromatase inhibitors (40.3%). The most commonly used aromatase inhibitor in this population was letrozole (96%), with anastrozole treating 4% of these patients. All the hormonal therapies (tamoxifen and aromatase inhibitors) were given as an adjuvant therapy. In addition to the hormonal therapies, 72.6% (45) of patients received radiotherapy and surgery, while 27.4% (17) of patients were treated with chemotherapy. Nonetheless, none of the patients were treated with targeted therapies among the enrolled breast cancer patients. Most of the patients who received chemotherapy had stage II and stage III breast cancer. Doxorubicin and cyclophosphamide combination therapy was the most (16, 94.1%) commonly used regimen among chemotherapy-treated patients.

### Comparison of the efficacy of treatment between tamoxifen and aromatase inhibitors

Among all stages of patients treated with tamoxifen, 48.6% (18) died, while among those treated with aromatase inhibitors, 40% (10) died. Among the 18 patients who died in the tamoxifen-treated group, 16 patients had late-stage breast cancer, while 2 patients had early-stage breast cancer. Among the 10 patients who died in the aromatase inhibitors treatment group, 6 of them had late-stage disease, while 4 patients had early-stage disease.

In the tamoxifen-treated group, 17 early-stage breast cancer patients showed a decrease in the tumor size while only 2 showed a reduction in the tumor size in advanced-stage breast cancer patients. Further, 8 advanced-stage breast cancer patients showed an increase in tumor size while 3 early-stage breast cancer patients showed an increase in the tumor size. In the aromatase inhibitor-treated group, 9 early-stage cancer patients showed a reduction in tumor size while only 6 advanced-stage patients had tumor size reduction. Moreover, 6 advanced-stage and 1 early-stage patient showed an increase in tumor size.

**Fig. 1:**
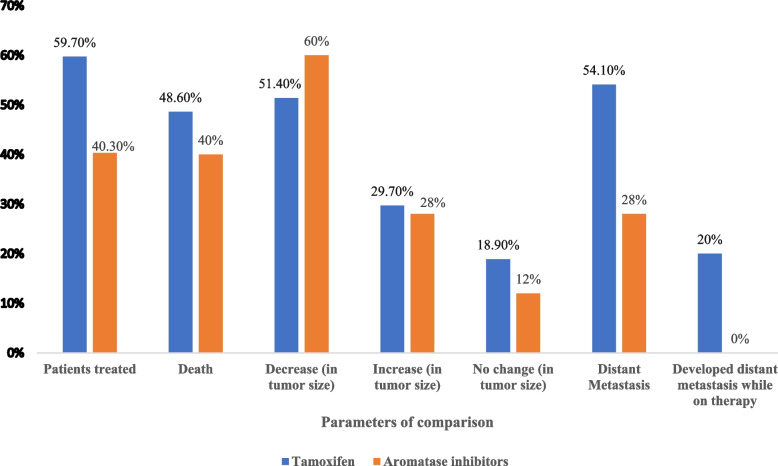
Comparison of the efficacy of tamoxifen and aromatase inhibitors

In all stages of breast cancer, patients on tamoxifen, 51.4% had a decrease in their tumor sizes compared with 60% of patients treated with aromatase inhibitors. On the other hand, 29.7% of patients treated with tamoxifen showed an increase in their tumor sizes compared with 28% of patients treated with aromatase inhibitors. In the tamoxifen group, 18.9% showed no change in tumor size, while 12% of patients showed no change in tumor size among the aromatase inhibitor group. Among the patients treated with tamoxifen, 54.1% (20) had distant metastasis, while 28% (7) had distant metastasis in the aromatase inhibitors group. Among the patients on tamoxifen who had distant metastasis, 20% (4) developed distant metastasis while on therapy, while none developed distant metastasis while on therapy for the aromatase inhibitors group (Fig. 1).

**Fig. 2:**
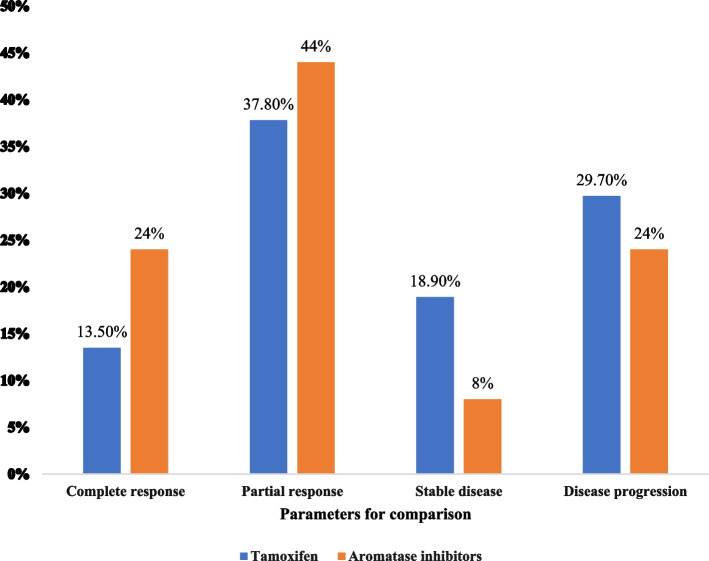
Patient response to treatment modalities

Among all stages of breast cancer patients treated with tamoxifen, 13.5%, 37.8%, 18.9%, and 29.7% showed a complete radiological response, partial radiological response, stable disease and disease progression, respectively. Among the patients treated with aromatase inhibitors, 24%, 44%, 8%, and 24% of patients showed complete response, partial response, stable disease and disease progression, respectively (Fig. 2). In early-stage breast cancer patients in the tamoxifen group, 5 patients showed complete radiological responses while none of the advanced-stage cancer in this group showed complete radiological responses. In the aromatase inhibitors groups, 4 early-stage and 2 advanced-stage patients showed complete response after treatment.

The study revealed that there was no significant difference in the median cancer-specific survival (*P* = 0.410), metastasis-free survival (*P* = 0.056) and survival after metastasis (*P* = 0.142). The tamoxifen group had a median survival time of 42.0 ± 3.6 months, while for aromatase inhibitors it was 49.0 months. The median survival from first radiologic metastasis to death was 6 ± 0.8 months in the group treated with tamoxifen and 8 ± 0.6 months in the aromatase group. The median metastasis-free survival for the tamoxifen group was 30.0 ± 1.4 months, while for the aromatase inhibitors group it was 42.0 months.

### Predictors of survival among breast cancer patients

A Cox regression analysis was done to determine the relationship between survival and the independent variables in the study (endocrine therapy, stage of cancer, menopausal status, tumor size, tumor grade, co-morbidities and age). There was a statistically significant association between stage IV disease, the presence of co-morbidities and survival in multivariate analyses. Stage IV breast cancer patients were 5.4 times (AOR = 5.4, 95% CI = 1.9–15.8, *P* = 0.002) more likely to die than stage II patients, while those with comorbidities were 3.8 times more likely to die (AOR = 3.8, 95% CI = 1.5–9.5, *P* = 0.005) than patients without comorbidities. Nonetheless, age, menopausal status, tumor size, tumor grade, and endocrine therapies were not significant predictors of survival (Table 3).

**Table 3:** Predictors of survival among breast cancer patients

Variable	Univariable analysis		Multivariable analysis	
	COR (95% CI)	*P*-value	AOR (95% CI)	*P*-value
Age				
≥ 50 years	1		1	
< 50 years	2.8(1.3–6.1)	0.009	4.3(0.9–19.4)	0.061
Stage of cancer				
Stage II	1		1	
Stage III	0.4(0.1–2.0)	0.278	0.5(0.1–2.5)	0.380
Stage IV	3.9(1.6–9.9)	0.004^*^	5.4(1.9–15.8)	0.002^*^
Menopausal status				
Premenopausal	1		1	
Post-menopausal	3.9(1.6–9.9)	0.678	1.6(1.4–4.8)	0.672
Tumor grade				
Low grade	1		1	
High grade	1.9(1.2–3.9)	0.947	1.4(1.3–5.8)	0.897
Tumor size				
Small (T1, ≤ 2 cm)	1		1	
Large (T2–T4, > 2 cm)	2.1(1.3–4.9)	0.547	2.2(1.4–6.7)	0.467
Comorbidity				
Absent	1		1	
Present	1.6(0.7–3.7)	0.254	3.8(1.5–9.5)	0.005^*^
Endocrine therapy				
Letrozole/Anastrozole	1		1	
Tamoxifen	1.3(0.6–3.0)	0.416	0.2(0.5–1.1)	0.074

*AOR* adjusted odds ratio,* COR* crude odds ratio, *CI* confidence interval

^*^Statistically significant *P*-value ≤ 0.05

## Discussion

In this study, the majority of patients (80.7%) had tumors that were both ER-positive and PR-positive, indicating a strong likelihood of responding to hormonal therapy. A smaller proportion of patients had tumors that were ER-positive but PR-negative (16.1%), while only 3.2% had PR-positive but ER-negative tumors. These variations suggest differing biological behavior and potential responsiveness to endocrine treatment.

In this study, most of the patients were classified as having Luminal A tumors, characterized by ER-positive, PR-positive, HER2-negative status and low Ki-67, typically associated with a favorable prognosis and good response to hormonal therapy. The remaining 16.1% were classified as Luminal B, defined by ER-positive, PR-negative, HER2-negative status and high Ki-67, which is generally more aggressive and may require combined endocrine and chemotherapy treatment.

In this study, letrozole has slightly better treatment outcomes than tamoxifen, both in terms of disease progression and survival. In this study, aromatase inhibitors showed slightly better than tamoxifen in terms of mortality reduction, 48.6% of patients on tamoxifen died compared to 40% on aromatase inhibitors. This is in line with results from a study showing that letrozole showed a greater reduction in breast cancer mortality in the aromatase group compared to the tamoxifen group by 2.1% [13]. In addition, other studies showed that the rate of mortality reduction in breast cancer patients treated with tamoxifen was lower than aromatase inhibitors [17–19]. According to a two-arm study done to compare aromatase inhibitors and tamoxifen, patients on letrozole and anastrozole both had better overall survival albeit being statistically insignificant [16]. This is consistent with this study, where the overall survival was better in the aromatase inhibitor group (49 months) compared to the tamoxifen group (42 months). In another study, in the letrozole group, the median overall survival time slightly exceeded that of the tamoxifen group, with durations of 34 months and 30 months, respectively [18]. After distant metastasis, the median survival time in this study was slightly better in the aromatase inhibitor group compared to the tamoxifen group by 2 months. This is similar to another study, whereby after metastasis, the median survival time was better in the aromatase inhibitor group compared to the tamoxifen group by 3 months [18].

In another study, it was noted that letrozole demonstrated superior tumor regression rates compared to tamoxifen [15]. Similar to this study, the findings in our setting revealed that 29.7% of patients in the tamoxifen group showed disease progression compared to 24% in the aromatase inhibitor group. Another study showed that aromatase inhibitors are more effective at slowing the time to disease progression by about 30% compared to tamoxifen [18]. In an article reviewing different trials around the world comparing the efficacy of aromatase inhibitors against tamoxifen in preventing distant metastasis, it was found that in all these trials there was a trend towards aromatase inhibitors being better at preventing distant metastasis [17]. Similarly, the findings in our setting depicted that 20% of patients who had distant metastasis developed distant metastasis while on therapy, while none developed distant metastasis while on aromatase inhibitors.

The present study showed that patients with co-morbid conditions were 3.8 times more likely to die compared to patients without comorbidities. In contrast, another study reported that the existence of comorbid conditions does not seem to correlate with the presence of more aggressive cancer types or distinct tumor biology characteristics [18]. Patients with comorbidities are less likely to undergo standard cancer treatments such as surgery, chemotherapy, and radiation therapy compared to those without comorbidities. Moreover, their chances of completing a full cancer treatment regimen are diminished, and certain studies suggest that this is associated with delayed cancer detection. There was also a statistically significant association between the stage of cancer and survival, with stage IV cancer patients being up to 5.4 times more likely to die than stage II breast cancer patients. Likewise, a previous study reported that stage IV breast cancer patients had up to 15.7% survival rate [20].

## Limitations of the study

Information on medication adherence and the exact duration of hormonal therapy was often not documented, limiting accurate assessment of treatment efficacy. The follow-up period is short, and hence late recurrences may be missed, especially in ER-positive patients. There may be no reliable data on whether patients took the medication as prescribed, which can impact efficacy outcomes. Furthermore, findings from a single-center setting may limit the generalizability of the findings to the general population. Data on lost to follow-up during the study period were not recorded which may introduce potential bias in survival estimates and outcome interpretations. Hence, future studies should have a robust follow-up system for accurate estimation of the outcomes. For patients who died outside the hospital, we were unable to confirm their death due to incomplete or outdated contact information.

## Conclusion

These findings collectively suggest a consistent trend towards improved treatment outcomes with aromatase inhibitors compared to tamoxifen. The observed reduction in mortality rates among aromatase inhibitor-treated patients highlights their potential clinical benefit, with superior overall survival and disease progression. Furthermore, the lower incidence of distant metastasis observed among patients receiving aromatase inhibitors suggests a potential role in preventing disease spread and improving long-term outcomes. However, further studies evaluating hormonal therapies and adjuvant treatments are crucial for reducing mortality rates and improving outcomes, addressing the disparity in breast cancer care between developed and developing regions.

## Data Availability

The data that support the findings of this study are available from the corresponding author upon reasonable request.
